# Supplementary material for: Polyketide synthase gene clusters in the ericoid mycorrhizal fungus Oidiodendron maius: genome-wide prediction, expression profiling, and functional characterization of knockout mutants
Source: Front Plant Sci. 2026 Sep 16;17:1924430. doi: 10.3389/fpls.2026.1924430 (PMC13623903; doi:10.3389/fpls.2026.1924430)
Supplement: Supplementary file 1 [file SupplementaryFile1.zip › Supplementary Materials/Supplementary Folder 1.zip!/Oidma1_AssemblyScaffolds_Repeatmasked/knownclusterblast/cluster46/jgi.p_Oidma1_135714_mibig_hits.html]

| MIBiG Protein | Description | MIBiG Cluster | MiBiG Product | % ID | % Coverage | BLAST Score | E-value |
| --- | --- | --- | --- | --- | --- | --- | --- |
| ABB90278.1 | heterokaryon\_incompatibility\_protein | BGC0001057 | Zearalenone\_biosynthetic\_gene\_cluster | 27 | 92.4332344214 | 165 | 2.3e-40 |
| EPE34326.1 | heterokaryon\_incompatibility\_protein | BGC0001035 | Pneumocandin\_biosynthetic\_gene\_cluster | 30 | 27.5964391691 | 84 | 4e-16 |
| EPE34353.1 | hypothetical\_protein | BGC0001035 | Pneumocandin\_biosynthetic\_gene\_cluster | 27 | 24.4807121662 | 61 | 3.6e-09 |
